# Editorial: One Health approach to mycobacterial infections in veterinary science

**DOI:** 10.3389/fvets.2026.1778469

**Published:** 2026-02-16

**Authors:** María Jimena Marfil, María Julia Traversa, Soledad Barandiaran, Ximena Ferrara Muñiz, Martín José Zumárraga, María Emilia Eirin

**Affiliations:** 1 Department of Large Animal Clinical Sciences, College of Veterinary Medicine, Michigan State University, East Lansing, MI, United States; 2 Área Medicina Preventiva, Laboratorio de Micobacterias, Dto. SAMP, Facultad de Ciencias Veterinarias, Universidad Nacional del Centro de la Provincia de Buenos Aires, CIVETAN, CIC, CONICET, Tandil, Argentina; 3 Instituto de Investigaciones en Producción Animal (INPA), Consejo Nacional de Investigaciones Científicas y Técnicas-Universidad de Buenos Aires, Buenos Aires, Argentina; 4 Servicio de Micobacterias, Centro de Vigilancia Sanitaria Veterinaria (VISAVET) Universidad Complutense Madrid, Madrid, Spain; 5 Instituto de Agrobiotecnología y Biología Molecular (IABiMo) UEDD CONICET-INTA, Centro de Investigación en Cs. Veterinarias y Agronómicas (CICVyA)-CNIA, Hurlingham, Argentina

**Keywords:** antimicrobial resistance, epidemiology and diagnosis, eradication and control, mycobacteria, One Health, vaccine, veterinary, zoonosis and reverse-zoonosis

Mycobacteria are ubiquitous pathogens that affect livestock, companion animals, wildlife, and humans, posing major challenges for veterinary medicine. Accurate diagnosis and targeted treatment are crucial to prevent antimicrobial resistance, yet integrated actions across the human–animal–environment interface remain limited, highlighting the need for a One Health approach (1, 2).

This Research Topic compiles 11 articles that advance the One Health understanding of veterinary-relevant mycobacteria by examining their epidemiology, diagnosis, treatment, zoonotic transmission dynamics, and by proposing integrated strategies to strengthen surveillance and disease control across interconnected systems.

Epidemiological studies traditionally use PCR, Sanger sequencing, spoligotyping, and MIRU-VNTR for mycobacterial typing. Although next-generation sequencing (NGS) provides higher resolution, conventional methods remain essential where NGS is unavailable. Rodríguez-Pazmiño et al. evaluated conserved genetic markers (16S*rRNA, hsp*65, *rpoB*) in 59 Non-tuberculous Mycobacteria (NTM) human isolates from Ecuador, concluding that concatenated 16S*rRNA-rpoB* sequencing offers a useful alternative when MALDI-ToF MS or WGS are unavailable, strengthening diagnostic capacity and surveillance in low- and middle-income settings (Rodriguez-Pazmiño et al.). Sandhu et al. demonstrated superior resolution of WGS over spoligotyping and VNTR in 3,052 *M. bovis* isolates from cattle and non-bovines in Great Britain. SNP-based classification enabled outbreak tracing in TB-free herds, benefiting animal health and farm management within a One Health framework. Matthews et al. detected pathogenic mycobacteria, including *M. bovis*, in rural KwaZulu-Natal water sources using multiple molecular methods. Although MTBC ecotypes differentiation remained incomplete, the findings underscore the need for further research in multi-host systems and highlight shared environmental reservoirs for livestock, wildlife, and humans (Matthews et al.). Hugh et al. reviewed *M. orygis*, reporting its rising incidence and emphasizing the need for advanced molecular tools to improve genomic characterization and surveillance. They stress the need for global, harmonized monitoring systems that account for regional One Health dynamics.

Accurate diagnosis is essential for identifying etiological agents. Agricultural expansion increases the risk of infections by poorly characterized mycobacteria living in natural ecosystems.

Emmerick et al. characterized four fast-growing isolates from Brazil's Atlantic Forest expressing immunogenic proteins shared with BCG and virulent *M. bovis* strains, raising concerns about diagnostic specificity and immune modulation. However, they did not use molecular tools, precluding definitive taxonomic classification (Emmerick et al.). This approach is relevant to mycobacterial taxonomy and to wildlife and human health, as these organisms can confound TB diagnosis in biodiverse regions. Diagnostic confirmation requires direct methods, as granulomas lack specificity. Orlando et al. addressed TB misdiagnosis in Ecuador, where TB-like lesions in humans and cattle are confirmed only by Ziehl–Neelsen (ZN) staining. Using smear staining, bacteriology, and multiplex qPCR on 795 bovines with TB-lesions detected in officially-inspected abattoirs, they identified six NTM and three non-Mycobacterial ZN-positive bacteria, but no *M. bovis* (Orlando et al.). These findings reinforce the need for a more accurate diagnosis and have direct implications for public health and veterinary services, as diagnostic errors affect zoonotic transmission tracking. Hugh et al. reported diagnostic limitations for *M. origys*, due to low MTBC diversity, although unique IS*6110* and DR region variations may improve diagnostic accuracy. Enhancing diagnostic tools will allow clarifications of transmission pathways shares by animals, humans and the environment.

Treatment of mycobacterial infections and resistance to anti-tuberculous agents remain critical challenges. Hugh et al. reviewed *M. orygis* therapy, noting scarce resistance data despite following standard MTBC regimens. Beyond surveillance of natural isolates, basic research provides insights into resistance mechanisms. Wang et al. characterized JTY_0672, a TetR-family regulator promoting *M. tuberculosis* survival and isoniazid resistance via increased triacylglycerol synthesis. Its overexpression in multidrug-resistant isolates suggests a potential therapeutic target benefiting One Health strategies.

Zoonotic and reverse-zoonotic transmission of *Mycobacterium* species poses global risks to disease control, food security, and trade. Ghielmetti et al. reported mixed *M. tuberculosis* infection (Lineages 1 and 4) in a captive African elephant in South Africa, presenting pneumonia and seropositivity. These findings highlight the need for measures to prevent reverse-zoonotic transmission in high-burden regions (Ghielmetti et al.). This case illustrates pathogen circulation between humans and wildlife in managed environments, requiring integrated veterinary–public health surveillance. Hugh et al. reviewed zoonotic transmission of *M. orygis*, identifying multiple human–animal pathways. In India, *M. orygis*-related TB exceeds *M. bovis* cases, and its rising global prevalence in Asia, spotlight its dual zoophilic and anthropophilic potential. These insights reinforce the need for cross-sector cooperation to detect emerging MTBC members capable of crossing species barriers.

Developing effective tools for mycobacteriosis control is critical to combat tuberculosis. BCG remains the only licensed vaccine, yet its limitations highlight the need for improved candidates, optimized administration strategies, and compatible diagnostics. Fernández et al. evaluated heat- and phage-inactivated *M. bovis, M. caprae*, and *M. microti* strains for protection and diagnostic interference in animal's experimental models. Heat-inactivated vaccines showed greater interference with PPD-B and P22 antigens than phage-inactivated ones, though all were compatible with defined antigen-based tests. Heat-inactivated *M. microti* (strain 16Z002093) exhibited the best overall performance (Fernández et al.). Cuenca-Lara et al. evaluated tuberculin test (TST) interference in goats vaccinated with BCG Danish strain 1331 and heat-inactivated *M. bovis* using intranasal and subcutaneous routes. A heterologous prime-boost regimen and the single-dose intranasal BCG achieved higher diagnostic specificity in TST and Interferon Gamma Release Assays (IGRA), while preserving cellular responses. Further evaluation under MTBC challenge conditions was recommended (Cuenca-Lara et al.). These strategies could improve TB control in livestock and reduce diagnostic cross-reactivity affecting trade and public health, while providing adaptable vaccination and diagnostic tools for diverse production systems.

Regionally adapted strategies for tuberculosis control in animal management are essential where conventional measures are impractical. Lakew et al. conducted a two- year study in a high-prevalence (98%) Ethiopian dairy herd. Early (≤5 days) segregation of newborn calves from infected dams produced disease-free young stock, with 76% remaining test-negative over 2 years, preserving valuable genetics. No clear link was found between dam test results and calf outcomes; highly reactive dams produced uninfected calves, and outcomes varied among offspring. These findings emphasize immediate post-birth separation without colostrum intake and the need for diagnostic tools beyond TST and IGRA to improve early detection (Lakew et al.). This study has One Health relevance by offering an actionable management strategy that reduces zoonotic risk, improves animal health and supports livelihoods of farming communities.

Contributions to this Research Topic underscored the need to advance molecular diagnostics, refine methodological frameworks, and adopt regionally adapted strategies to improve surveillance and control mycobacterial diseases. By integrating humans, animals and environment data, this Research Topic reinforces a coordinated One Health foundation for mitigating mycobacterial threats globally.
